# Menopausal Status Combined with Serum CA125 Level Significantly Predicted Concurrent Endometrial Cancer in Women Diagnosed with Atypical Endometrial Hyperplasia before Surgery †

**DOI:** 10.3390/diagnostics12010006

**Published:** 2021-12-21

**Authors:** Yaochen Lou, Jiongbo Liao, Weiwei Shan, Zhiying Xu, Xiaojun Chen, Jun Guan

**Affiliations:** 1 Department of Gynecology, Obstetrics and Gynecology Hospital of Fudan University, Shanghai 200011, China; yclou14@fudan.edu.cn (Y.L.); liaojiongbo7405@fckyy.org.cn (J.L.); danweiwei7468@fckyy.org.cn (W.S.); xuzhiying7911@fckyy.org.cn (Z.X.); 2 Shanghai Key Laboratory of Female Reproductive Endocrine Related Diseases, Shanghai 200011, China

**Keywords:** atypical endometrial hyperplasia, endometrial cancer, postmenopausal status, cancer antigen 125

## Abstract

About 10–66% of patients with atypical endometrial hyperplasia diagnosed before surgery (preoperative-AEH) are found to have concurrent endometrial cancer (EC) at definitive hysterectomy, leading to incomplete primary surgery and delayed adjuvant treatment. This study aims to investigate the potential risk factors of concurrent EC in preoperative-AEH patients in a clinical setting with a gynecological pathology review. All patients diagnosed with AEH by endometrial biopsy or curettage that then underwent definitive hysterectomy from January 2016 to December 2019 in a tertiary hospital were retrospectively analyzed. All diagnoses were reviewed by gynecological pathologists. A total of 624 preoperative-AEH patients were included, 30.4% of whom had concurrent EC. In multivariate analysis, postmenopausal status and CA125 ≥ 35 U/mL significantly correlated with concurrent EC (OR = 3.57; 95% CI = 1.80–7.06; OR = 2.15; 95% CI = 1.15–4.03). This risk was remarkably increased in patients with both postmenopausal status and CA125 ≥ 35 U/mL (OR = 16.20; 95% CI = 1.73–151.44). Notably, concurrent EC seemed to occur more frequently in women with postmenopausal time ≥ 5 years (OR = 4.04, 95% CI = 1.80–5.85). In addition, CA125 ≥ 35 U/mL seemed to be an independent risk factor (OR = 5.74; 95% CI = 1.80–18.27) for concurrent intermediate-high-risk EC. Intermediate-high-risk EC was also more commonly seen in preoperative-AEH women with postmenopausal time ≥ 5 years (OR = 5.52, 95% CI = 1.21–25.19, *p* = 0.027). In conclusion, preoperative-AEH patients with postmenopausal status or elevated level of CA125 might have a high risk of concurrent EC. Adequate pre-surgical evaluation might be suggested for such patients.

## 1. Introduction

Atypical endometrial hyperplasia (AEH), a precancerous lesion of endometrial cancer (EC), is a hyperplasic state of the endometrium with nuclear atypia based on pathological findings [1,2]. The standard treatment for AEH is hysterectomy with bilateral salpingectomy. However, of patients preoperatively diagnosed with AEH (preoperative-AEH patients) by endometrium biopsy or curettage, about 10–66% were demonstrated to have co-existing EC at definitive hysterectomy [3,4,5,6,7,8,9,10]. According to National Comprehensive Cancer Network (NCCN) guidelines (version 1.2021), for patients with EC found by incompletely surgically stage, the risk stratification should be assessed [11]. In this context, for those patients with high-risk factors, imaging or surgical restaging should have been recommended, and if necessary, a second surgery was then required to assess lymph node metastasis to guide adjuvant therapy [11]. Although the sentinel lymph node (SLN) sampling is widely accepted with fewer adverse events than traditional systematic lymphadenectomy, it is impossible for preoperative-AEH patients who had undergone hysterectomy due to the disruption of the lymphatic channels. Thus, it is clinically important to identify EC patients who were initially diagnosed with AEH before surgery, to avoid the risk of second anesthesia and multiple side effects caused by systematic lymphadenectomy.

EC-related risk factors were found, including chronic anovulation, obesity, metabolic disorders (e.g., diabetes, hypertension), exogenous estrogen exposure, nulliparity, late-onset menopause, and older age, etc., [12,13,14]. At present, no consensus had been reached on the factors predicting concurrent EC in preoperative-AEH patients, or risk stratification for coexisting EC before hysterectomy [5,10,15,16]. That may be due to the lack of pathological central review on AEH specimens before surgery, or relatively small sample size (mostly <200 cases) in published studies. A large (*n* = 773) retrospective study based on the Danish Gynecological Cancer Database reported that age and menopause were significantly correlated with EC risk in preoperative-AEH patients [5]. The author also mentioned that the findings might not be suitable for clinical use as the study focused on a community setting where different pathologists assessed the specimens without an expert pathology review [5]. A recent study analyzed 169 women with complex atypical hyperplasia before surgery in a tertiary hospital and showed that age and endometrial stripe thickness ≥2 cm were the strongest predictors of concurrent EC at the time of hysterectomy [15]. Considering the difficulty in distinguishing AEH and well-differentiated endometrial adenocarcinoma in surgical pathology, large-scale research with central review by pathological specialists is needed to better evaluate potential risk factors of EC in preoperative-AEH cases.

In this context, we conducted a retrospective study to investigate which preoperative factors might be correlated with concurrent EC in a large cohort of preoperative-AEH patients (*n* = 624) from a tertiary obstetrics and gynecology hospital with a pathological central review. The second objective was to assess which preoperative-AEH patients might be at risk of intermediate-high-risk EC in the final diagnosis as they might need more clinical assessment.

## 2. Materials and Methods

### 2.1. Patient Cohort

A total of 655 consecutive patients diagnosed with AEH preoperatively were retrospectively included in this study. All patients underwent definitive hysterectomy in the Obstetrics and Gynecology Hospital of Fudan University (Ob& Gyn Hospital) from January 2016 to December 2019. The inclusion criteria were patients who (1) were diagnosed with AEH preoperatively by endometrium biopsy or curettage; 92) underwent definitive hysterectomy within 1 month after AEH diagnosis; (3) had no other malignant tumors; (4) received no previous fertility-sparing therapies before hysterectomy; and (5) had available clinicopathological data. Thirty-one patients were excluded for receiving progestin treatment before hysterectomy. Therefore, totally 624 women met the criteria and then were recruited into the study. All the patients diagnosed with endometrial cancer after definitive surgery were staged according to the International Federation of Gynecology and Obstetrics (FIGO) 2009 staging system [17]. The study was approved by the Ethics Committees of the Ob& Gyn Hospital (protocol code 2021-185). All patients had signed informed consent forms for using their clinicopathological data for research purposes.

### 2.2. Pathological Diagnosis

All 624 patients were diagnosed with AEH only in Ob& Gyn Hospital through endometrial biopsy by Pipelle or dilation and curettage (D&C) with or without hysteroscopy (HSC). Final pathological diagnoses were made after definitive hysterectomy. Pathological diagnoses were performed according to the World Health Organization (WHO) pathological classification (2014) and were determined by at least two senior gynecological pathologists only in Ob& Gyn Hospital. If the diagnosis differed, a consultation would be held in the department of pathology for the final decision.

Patients diagnosed with EC after definitive surgery were classified with low-risk EC or intermediate-high-risk EC. In this study, low-risk EC was defined as: endometrioid endometrial cancer grade 1–2, myometrial invasion <50% and no other risk factors presented (which included grade 3, non-endometrioid endometrial cancer, myometrial invasion ≥50%, cervical stromal involvement, extra-uterine involvement or lymph-vascular space invasion (LVSI)). All other endometrial cancer cases were defined as intermediate-high-risk EC [11].

### 2.3. Data Collection

Clinical and pathological data were collected by experienced clinicians, including age, weight, height, menopausal status, fertility status, medical history, comorbidities (hypertension and diabetes), ultrasound evaluation and endometrium biopsy methods (Pipelle or D&C, with or without HSC). Laboratory data including fasting blood glucose (FBG), fasting insulin (FINS) and serum level of cancer antigen 125 (CA125) were also recorded. Clinical data were collected before definitive surgery. Pathological reports from endometrial biopsy and hysterectomy were collected.

Body mass index (BMI) and the homeostasis model assessment-insulin resistance (HOMA-IR) index were calculated based on following formula: (1) BMI = weight (kg)/height (m^2^); (2) HOMA-IR = FBG (mmol/L) × FINS (mU/L)/22.5. Patients with BMI ≥ 28 (kg/m^2^) were defined as obese [18], whereas patients with HOMA-IR ≥ 2.95 were defined as insulin resistant, according to previous studies [19,20].

### 2.4. Statistical Analysis

Statistical analysis was performed by SPSS 25.0 (SPSS, Chicago, IL, USA). The Shapiro-Wilk test or Kolmogorov–Smirnov test was used as appropriate to ascertain whether continuous variables had a normal distribution. Homogeneity of variance was analyzed by Levene’s test. Continuous variables were summarized by medians and interquartile range, and categorical variables were presented as frequency with percentage. The intra-group differences of continuous variables were investigated by Student’s *t* test when normally distributed, or Mann–Whitney U test when non-normally distributed. Chi-squared test or Fisher’s exact was used to analyze the difference between categorical variables as appropriate. Logistic regression models were used for univariate and multivariate statistical comparisons. Factors found significant in univariate analysis or with clinical importance were included into multivariate analysis. Adjusted odds ratio (OR) and 95% confidence intervals (CIs) were estimated with the logistic regression models. A 2-tailed *p* value of less than 0.05 was considered statistically significant.

As only 447 of 624 women had CA125 value, the analyses on the correlation between CA125 and concurrent EC were only performed on these patients.

## 3. Results

Of 624 patients who met the inclusion criteria, 434 (69.6%) maintained AEH in the final diagnosis, whereas 190 (30.4%) had co-existing EC, including 160 (25.6%) patients with low-risk EC and 30 (4.8%) with intermediate-high-risk EC, respectively. Basic characteristics of the patients were presented in Table 1, Tables S1 and S2. Most of patients with EC diagnosed by final histopathology (final-EC) were with FIGO stage IA (89.5%), grade 1 (94.0%), myometrial invasion <50% (68.9%), endometrioid histology (96.8%) and absence of LVSI (94.7%, Table S1). Only 447 women had CA125 value, and they were included in the analysis of the correlation between CA125 and concurrent EC. The median age in the final-AEH and final-EC group were 47 and 49 years old. The median BMI, the distribution of diabetes and hypertension, and endometrial sampling methods before surgery were similar between the two groups. 

Compared with patients maintained AEH in final diagnosis, those with concurrent EC seemed to be slightly older (*p* = 0.002), with higher level of FBG (*p* = 0.039) and CA125 (*p* = 0.033), and higher percentage of postmenopausal status (*p* < 0.001, Table 1). 

Univariate and multivariate analyses were carried out to investigate possible risk factors predicting concurrent EC in final histopathology (Table 2). Univariate analysis showed postmenopausal status and CA125 ≥ 35 U/mL were correlated with concurrent EC. In multivariate analysis, CA125 level was not included into the model first because only 447 of 624 patients had this information. Multivariate analysis in all 624 patients showed postmenopausal status was independently correlated with concurrent EC (OR = 3.17; 95% CI = 1.85–5.46; *p* < 0.001), adjusted for age, BMI, diabetes, hypertension and FBG level. When analyzing 447 patients with available CA125 value, both postmenopausal status (OR = 3.57; 95% CI = 1.80–7.06; *p* < 0.001) and CA125 ≥ 35 U/mL (OR = 2.15; 95% CI = 1.15–4.03; *p* = 0.017) as well as hypertension (OR = 1.70; 95% CI = 1.00–2.87; *p* = 0.049) were found to be independent risk factors for finally diagnosed EC, after adjusting age, BMI, diabetes and FBG level (Table 2).

**Figure 1:**
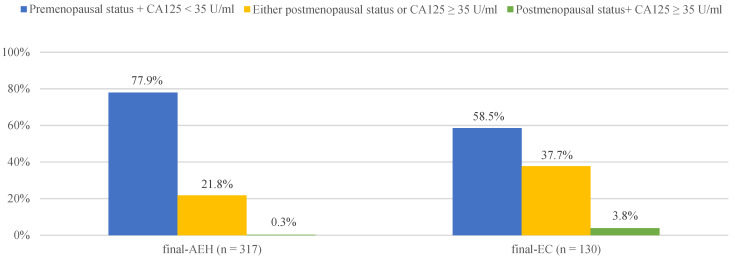
The distribution of final-AEH and final-EC patients diagnosed postoperatively in 3 subgroups according to menopausal status and CA125 value preoperatively. Notes: According to menopause status and serum of CA125 level, patients were divided into three subgroups: “premenopausal status + CA125 <3 5 U/mL”, “either postmenopausal status or CA125 ≥ 35 U/mL” and “postmenopausal status + CA125 ≥ 35 U/mL”. Abbreviations: final-AEH, atypical endometrial hyperplasia diagnosed by final histopathology; final-EC, endometrial cancer diagnosed by final histopathology; CA125, cancer antigen 125.

Furthermore, we found 3.8% (5/130), 37.7% (49/130) and 58.5% (76/130) of finally diagnosed EC patients had “postmenopausal status + CA125 ≥ 35 U/mL” (both risk factors), “either postmenopausal status or CA125 ≥ 35 U/mL” (either one risk factor) and “premenopausal status + CA125 < 35 U/mL” (no risk factor), compared with 0.3% (1/317),21.8% (69/317) and 77.9% (185/317) in finally diagnosed AEH patients, respectively (Figure 1). Preoperative-AEH women with “postmenopausal status + CA125 ≥ 35 U/mL” seemed to have remarkably increased risk for concurrent EC (OR = 16.20; 95% CI = 1.73–151.44; *p* = 0.015). The positive predictive value of “postmenopausal status + CA125 ≥ 35 U/mL” for concurrent EC was 83.3% (5/6) while the negative predictive value was 71.7% (316/441) (Table S3).

We then looked at possible risk factors correlated with concurrent intermediate-high-risk EC in preoperative-AEH patients. Only CA125 ≥ 35 U/mL was found to be the independent and significant predictor in both uni- and multivariate analyses (OR = 4.54; 95% CI = 1.58–13.04; *p* = 0.005, OR = 5.74; 95% CI = 1.80–18.27; *p* = 0.003, Table 3). 

We further asked whether longer time after menopause was correlated with a higher risk of co-existing EC. We divided patients into 4 subgroups: premenopausal, postmenopausal time < 2 years, ≥2–<5 years, and ≥5 years (Figure S1). Compared with premenopausal group, the risk of concurrent EC was higher in postmenopausal women but remain stable within the first 5 years after menopause (<2 years: OR = 2.87, 95% CI = 1.20–6.84, *p* = 0.018; and ≥2–<5 years: OR = 2.95, 95% CI = 1.47–5.91, *p* = 0.002, Table 4). Nevertheless, this risk continued to increase after 5 years since menopause (OR = 4.04, 95% CI = 1.80–5.85, *p* = 0.001, Table 4). A similar result was also observed when including CA125 level into the analysis model. The risk of concurrent EC in preoperative-AEH patients with postmenopausal time ≥5 years was almost 6-fold higher than that in premenopausal group (OR = 6.35, 95% CI = 2.37–17.03, *p* < 0.001, Table 4). Notably, we found the probability to have intermediate-high-risk EC was only elevated in women with postmenopausal time ≥ 5 years (OR = 5.52, 95% CI = 1.21–25.19, *p* = 0.027), with no difference in this risk among the other three groups (Table 5). Similar result was also found when including CA125 level into the analysis model (OR = 8.06, 95% CI = 1.14–56.83, *p* = 0.036, Table 5).

## 4. Discussion

The present study investigated the potential risk factors of concurrent EC in a large amount (*n* = 624) of preoperative-AEH patients. In a clinical setting with an expert pathological review, postmenopausal status and CA125 ≥ 35 U/mL were both independent and strong predictors. Particularly, the risk for concurrent EC was remarkably increased in preoperative-AEH patients with the combination of postmenopausal status and CA125 ≥ 35 U/mL (OR = 16.20; 95% CI = 1.73–151.44). Notably, among all postmenopausal AEH women, the risk of concurrent EC seemed to be further increased after 5 years since menopause.

Despite several correlational research, no consensus has been currently reached on risk factors of final-diagnosed EC in preoperative-AEH women [5,10,15,16]. Compared with previous findings, we conducted a large-scale study in a tertiary hospital with good quality of pathology review to more accurately analyze the predictors of concurrent EC, in order to help screen high-risk patients for more appropriate clinical assessment and surgical strategy.

All the specimens in our study were reviewed by gynecological pathologists in a single tertiary hospital to ensure the consistency and high quality of the diagnosis. That might be the reason why relatively fewer preoperative-AEH patients (30.4%) in our study had concurrent EC, compared with 40–60% in most published articles [5,8,21]. The distinction between AEH and well-differentiated EC is difficult in pathology. First, the application of different histologic criteria (WHO 2014 or Endometrial Intraepithelial Neoplasia criteria) and various thresholds could lead to different pathological diagnoses [1,22,23]. Second, it is hard to differentiate the histologic features of muscular invasion that was an important part to diagnose EC [23]. In addition, technical issues and subjectivity could influence the diagnosis, including insufficient clinical data, inadequate sampling, unsuitable fixation and insufficient staining quality [1,2,22]. The pathologists in our obstetrics and gynecology hospital were experienced in evaluating endometrium tissues, which might reduce the bias in diagnosis when evaluating the risk factor of concurrent EC.

We reported the significant correlation between menopause and concurrent EC of preoperative-AEH patients in a clinical setting. Similar findings had been presented in a large community-based study including 773 preoperative-AEH women from the Danish Gynecological Cancer Database (DGCD) [5]. It showed that 80% of postmenopausal women had an almost 3-fold higher risk of final diagnosed EC compared with the premenopausal group [5]. The researcher also explained that it was a “real-world” study and might not be suitable for clinical evaluation, as it lacked hospital-based information and the surgical specimens were assessed by different pathologists without expert pathology review [5]. Our study further supported the predictive value of postmenopausal status on concurrent EC in a tertiary hospital with gynecological pathology review. Additionally, among all postmenopausal women diagnosed with AEH before surgery, we found those with menopause time > 5 years had the highest risk of concurrent EC, which might provide more practical information for clinical decision making.

Except for menopause, CA125 ≥ 35 U/mL was also found as a predictor for both concurrent EC and intermediate-high-risk EC in our study. As a glycoprotein originating from the coelomic epithelium including endometrium, CA125 level was observed physiologically lower when healthy women come to menopause because of the atrophic endometrium or concomitant low gonadal steroid levels [24,25]. Conversely, in cancerous endometrium, CA125 levels could increase even when the patients were postmenopausal because endometrial tumor cells could directly promote the synthesis of CA125 [25,26]. In addition, aromatic androgens increased in EC cells could be converted to estrogens and then caused an increased level of CA125 [25,27]. In this study, we defined CA125 ≥ 35 U/mL as a cut-off value, because it has been widely accepted in most medical centers by chemiluminescent immunoassay with detection standard “0–35 U/mL” [28,29,30]. Sood et al. found that CA125 was one of the most powerful predictors of overall survival in EC patients, and those EC women with CA125 ≥ 35 U/mL had a significantly worse 5-year survival rate [31]. Consistent with previous findings, CA125 ≥ 35 U/mL seemed to be a good predictor for concurrent EC in our study, nonetheless, further verifications in prospective studies are still needed.

Our findings are of clinical significance because they could facilitate the pre-surgical evaluation and decision making for preoperative-AEH patients. Even with the pathological central review, there were still 30.4% of preoperative-AEH women in our study with concurrent EC, including 4.8% with intermediate-high-risk EC. In this situation, those patients with undiagnosed EC, particularly with the intermediate-high-risk EC, could benefit from pre-surgical pelvic magnetic resonance imaging (MRI) evaluation and comprehensive staging surgery. According to our results, preoperative-AEH patients with either predictor (menopause and CA125 ≥ 35 U/mL) may need adequate pre-surgical assessment due to the much-increased cancer risk and potential unfavorable prognosis. Additionally, both predictive markers (postmenopausal status and CA125 ≥ 35 U/mL) are easy to measure; therefore, it could be involved in clinical routine assessment for AEH women diagnosed before surgery.

The study was with some limitations. First, it was a retrospective study conducted in a single tertiary hospital. In addition, only 447 out of 624 patients had serum CA125 value which was a major limitation. Nevertheless, whether including or excluding the CA125 value into multivariate analyses, postmenopausal status remained significant in predicting concurrent EC. When including the CA125 value into the analysis model, the sample size was still large (*n* = 447) to conclude convincing results that patients with CA125 ≥35 U/mL might be more likely to progress from AEH to EC in the final diagnosis. The wide confidence intervals of “postmenopausal status + CA125 ≥ 35 U/mL” may be due to the small number of samples. Thus, further prospective studies on predictive value of these markers are needed.

In conclusion, for preoperative-AEH women with postmenopausal status or serum CA125 ≥35 U/mL, expert pathological review and comprehensive pre-surgical assessment in tertiary centers might be suggested. Further verifications on these markers in prospective studies are required to provide optimal clinical-decisions and patient-management in the future.

## Figures and Tables

**Table 1:** Clinical characteristics of patients diagnosed with AEH and EC by final histopathology.

Characteristics	Number	Total (*n* = 624)	Final-AEH (*n* = 434)	Final-EC (*n* = 190)	*p* Value ^b,c^
Median (interquartile range)					
Age (years old)	624	48 (43–51)	47 (43–51)	49 (44–53)	0.002
BMI (kg/m^2^) ^a^	621	24.61 (22.45–27.34)	24.61 (22.51–27.34)	24.46 (22.34–27.60)	0.979
Number (%)					
BMI (kg/m^2^) ^a^	621				0.860
<28		489 (78.7%)	341 (78.9%)	148 (78.3%)	
≥28		132 (21.3%)	91 (21.1%)	41 (21.7%)	
Menopausal status	624				<0.001
Premenopausal		506 (81.1%)	374 (86.2%)	132 (69.5%)	
Postmenopausal		118 (18.9%)	60 (13.8%)	58 (30.5%)	
Fertility	624				0.235
Pluripara		597 (95.7%)	418 (96.3%)	179 (94.2%)	
Nullipara		27 (4.3%)	16 (3.7%)	11 (5.8%)	
Tubal ligation	624				0.335
NO		564 (90.4%)	389 (89.6%)	175 (92.1%)	
YES		60 (9.6%)	45 (10.4%)	15 (7.9%)	
Diabetes	624				0.447
NO		588 (94.2%)	411 (94.7%)	177 (93.2%)	
YES		36 (5.8%)	23 (5.3%)	13 (6.8%)	
Hypertension	624				0.081
NO		484 (77.6%)	345 (79.5%)	139 (73.2%)	
YES		140 (22.4%)	89 (20.5%)	51 (26.8%)	
FBG (mmol/L)	607				0.039
<7.0		580 (95.6%)	409 (96.7%)	171 (92.9%)	
≥7.0		27 (4.4%)	14 (3.3%)	13 (7.1%)	
HOMA-IR ^a^	246				0.186
<2.95		196 (79.7%)	151 (81.6%)	45 (73.8%)	
≥2.95		50 (20.3%)	34 (18.4%)	16 (26.2%)	
CA125 (U/mL) ^a^	447				0.033
<35		397 (88.8%)	288 (90.9%)	109 (83.8%)	
≥35		50 (11.2%)	29 (9.1%)	21 (16.2%)	
Sampling method	624				0.421
D&C alone		428 (68.6%)	294 (67.7%)	134 (70.5%)	
D&C with HSC		189 (30.3%)	133 (30.6%)	56 (29.5%)	
Pipelle biopsy		7 (1.1%)	7 (1.6%)	0 (0%)	

Data shown were median (interquartile range) or number (%). ^a^ All variables were analyzed among 624 patients except for BMI, FBG, HOMA-IR and CA125. Missing data included 3 cases for BMI, 17 for FBG, 378 for HOMA-IR and 177 for CA125. ^b^ *p* value: difference between final-AEH group and final-EC group. ^c^ Significant difference *p* < 0.05. Abbreviations: AEH, atypical endometrial hyperplasia; EC, endometrial cancer; final-AEH, atypical endometrial hyperplasia diagnosed by final histopathology; final-EC, endometrial cancer diagnosed by final histopathology; BMI, body mass index; FBG, fasting blood glucose; HOMA-IR, homeostasis model assessment-insulin resistance; CA125, cancer antigen 125; D&C, dilatation and curettage; HSC, hysteroscopy.

**Table 2:** Univariate and multivariate analyses of factors predicting concurrent EC in final histopathology for 624 preoperative-AEH patients ^a^ and 447 preoperative-AEH patients ^b^ with available serum CA125 value according to logistic regression model.

Univariate Analysis					Multivariate Analysis without Available CA125 ^f^					Multivariate Analysis with Available CA125 ^g^				
**Characteristics**	**No.**	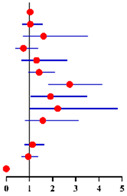	**OR (95% CI)**	***p*** **^e^**	**Characteristics**	**No.**	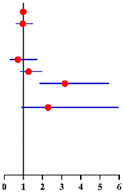	**Adjusted OR ^c^ (95% CI)**	***p*** **^e^**	**Characteristics**	**No.**	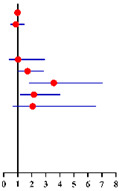	**Adjusted OR ^d^ (95% CI)**	***p*** **^e^**
**Age (years)**:	624		1.03 (1.01–1.06)	0.007	**Age (years)**:	624		0.99 (0.96–1.02)	0.574	**Age (years)**:	447		0.98 (0.94–1.02)	0.275
**BMI (kg/m²)**: ≥28 vs. <28	621		1.04 (0.69–1.57)	0.860	**BMI (kg/m²)**: ≥28 vs. <28	621		0.97 (0.61–1.52)	0.879	**BMI (kg/m²)**: ≥28 vs. <28	446		0.86 (0.49–1.49)	0.582
**Fertility**: Nullipara vs. Pluripara	624		1.61 (0.73–3.53)	0.239				/	/				/	/
**Tubal ligation**: Yes vs. No	624		0.74 (0.40–1.37)	0.336				/	/				/	/
**Diabetes**: Yes vs. No	624		1.31 (0.65–2.65)	0.448	**Diabetes**: Yes vs. No	624		0.71 (0.29–1.74)	0.452	**Diabetes**: Yes vs. No	447		1.02 (0.35–2.94)	0.975
**Hypertension**: Yes vs. No	624		1.42 (0.96–2.11)	0.082	**Hypertension**: Yes vs. No	624		1.27 (0.81–1.98)	0.302	**Hypertension**: Yes vs. No	447		1.70 (1.00–2.87)	**0.049**
**Menopausal status**: Post vs. Pre	624		2.74 (1.81–4.14)	**<0.001**	**Menopausal status**: Post vs. Pre	624		3.17 (1.85–5.46)	**<0.001**	**Menopausal status**: Post vs. Pre	447		3.57 (1.80–7.06)	**<0.001**
**CA125 (U/ml)**: ≥35 vs. <35	447		1.91 (1.05–3.50)	**0.035**				/	/	**CA125 (U/ml)**: ≥35 vs. <35	447		2.15 (1.15–4.03)	**0.017**
**FBG (mmol/L)**: ≥7.0 vs. <7.0	607		2.22 (1.02–4.82)	**0.044**	**FBG (mmol/L)**: ≥7.0 vs. <7.0	607		2.29 (0.88–5.98)	0.090	**FBG (mmol/L)**: ≥7.0 vs. <7.0	435		2.06 (0.65–6.58)	0.221
**HOMA-IR**: ≥2.95 vs. <2.95	246		1.58 (0.80–3.12)	0.189				/	/				/	/
Sampling method:														
**D&C alone**: Yes vs. No	624		1.14 (0.79–1.65)	0.491				/	/				/	/
**D&C with HSC**: Yes vs. No	624		0.95 (0.65–1.37)	0.769				/	/				/	/
**Pipelle biopsy**: Yes vs. No	624		0.00 (0.00)	0.999				/	/				/	/
Final diagnosis					Final diagnosis					Final diagnosis				
		← AEH EC →					← AEH EC →					← AEH EC →		

^a^ In total of 624 patients with available data. ^b^ In total of 447 patients who had serum CA125 value with available data. ^c^ OR adjusted for age, BMI, diabetes, hypertension and FBG level. ^d^ OR adjusted for age, BMI, diabetes and FBG level. ^e^ Significant difference *p* < 0.05. Notes: ^f^ CA125 was not included into this analysis for only 447 of 624 preoperative-AEH patients had available serum CA125 value. ^g^ This analysis was performed in 447 of 624 preoperative-AEH patients with available serum CA125 value. Abbreviations: AEH, atypical endometrial hyperplasia; EC, endometrial cancer; OR, odds ratio; CI, confidence interval; BMI, body mass index; CA125, cancer antigen 125; FBG, fasting blood glucose; HOMA-IR, homeostasis model assessment—insulin resistance; D&C, dilatation and curettage; HSC, hysteroscopy.

**Table 3:** Univariate and multivariate analyses of factors related to concurrently intermediate-high-risk EC in 190 final-EC patients ^a^ and in 130 final-EC patients ^b^ with available serum CA125 value according to logistic regression model.

Univariate Analysis					Multivariate Analysis without Available CA125 ^f^					Multivariate analysis with available CA125 ^g^				
**Characteristics**	**No.**	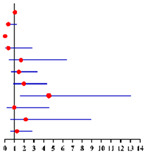	**OR (95% CI)**	***p*** **^e^**	**Characteristics**	**No.**	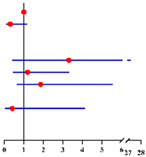	**Adjusted OR ^c^ (95% CI)**	***p*** **^e^**	**Characteristics**	**No.**	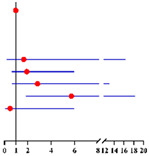	**Adjusted OR ^d^ (95% CI)**	***p*** **^e^**
**Age (years)**:	190		1.05 (0.99–1.10)	0.096	**Age (years)**:	190		1.01 (0.93–1.08)	0.884	**Age (years)**:	130		0.97 (0.89–1.06)	0.530
**BMI (kg/m²)**: ≥28 vs. <28	189		0.35 (0.10–1.23)	0.103	**BMI (kg/m²)**: ≥28 vs. <28	189		0.31 (0.08–1.17)	0.084				/	/
**Fertility**: Nullipara vs. Pluripara	190		0 (0)	0.999				/	/				/	/
**Tubal ligation**: Yes vs. No	190		0.36 (0.05–2.84)	0.332				/	/				/	/
**Diabetes**: Yes vs. No	190		1.67 (0.43–6.45)	0.460	**Diabetes**: Yes vs. No	190		3.30 (0.40–27.21)	0.267	**Diabetes**: Yes vs. No	130		1.66 (0.17–16.38)	0.664
**Hypertension**: Yes vs. No	190		1.45 (0.63–3.36)	0.384	**Hypertension**: Yes vs. No	190		1.20 (0.43–3.34)	0.733	**Hypertension**: Yes vs. No	130		1.90 (0.61–5.99)	0.270
**Menopausal status**: Post vs. Pre	190		1.95 (0.88–4.35)	0.101	**Menopausal status**: Post vs. Pre	190		1.86 (0.62–5.56)	0.265	**Menopausal status**: Post vs. Pre	130		2.83 (0.62–12.96)	0.180
**CA125 (U/mL)**: ≥35 vs. <35	130		4.54 (1.58–13.04)	**0.005**				/	/	**CA125 (U/ml)**: ≥35 vs. <35	130		5.74 (1.80–18.27)	**0.003**
**FBG (mmol/L)**: ≥7.0 vs. <7.0	184		0.97 (0.20–4.62)	0.969	**FBG (mmol/L)**: ≥7.0 vs. <7.0	184		0.41 (0.04–4.13)	0.448	**FBG (mmol/L)**: ≥7.0 vs. <7.0	127		0.49 (0.04–5.98)	0.580
**HOMA-IR**: ≥2.95 vs. <2.95	61		2.17 (0.52–8.97)	0.286				/	/				/	/
**Sampling method**: D&C with HSC vs. D&C alone	190		1.24 (0.54–2.85)	0.614				/	/				/	/
Final diagnosis					Final diagnosis					Final diagnosis				
	← low-risk EC intermediate-high-risk EC →					← low-risk EC intermediate-high-risk EC →					← low-risk EC intermediate-high-risk EC →			

^a^ In total of 190 patients with available data. ^b^ In total of 130 patients who had serum CA125 value with available data. ^c^ OR adjusted for age, BMI, diabetes, hypertension, menopausal status and FBG level. ^d^ OR adjusted for age, diabetes, hypertension, menopausal status and FBG level. ^e^ Significant difference *p* < 0.05. Notes: ^f^ CA125 was not included into this analysis for only 130 of 190 final-EC patients had available serum CA125 value. ^g^ This analysis was performed in 130 of 190 final-EC patients with available serum CA125 value. Abbreviations: EC, endometrial cancer; final-EC, endometrial cancer diagnosed by final histopathology; OR, odds ratio; CI, confidence interval; BMI, body mass index; CA125, cancer antigen 125; FBG, fasting blood glucose; HOMA-IR, homeostasis model assessment—insulin resistance; D&C, dilatation and curettage; HSC, hysteroscopy. Low-risk EC postoperatively was defined as: endometrioid endometrial cancer grade 1–2, myometrial invasion < 50% and no other risk factors presented (which included grade 3, non-endometrioid endometrial cancer, myometrial invasion ≥ 50%, cervical stromal involvement, extra-uterine involvement or lymph-vascular space invasion). All other endometrial cancer cases were defined as intermediate-high-risk EC.

**Table 4:** Multivariate logistic analysis models on the correlation between menopausal statuses (premenopausal status, postmenopausal time <2 years, ≥2–<5 years and ≥5 years) and concurrent EC in final histopathology for 624 preoperative-AEH patients ^a^ and 447 preoperative-AEH patients ^b^ with available serum CA125 value.

Multivariate Analysis without Available CA125 ^f^					Multivariate Analysis with Available CA125 ^g^				
**Characteristics**	**No.**	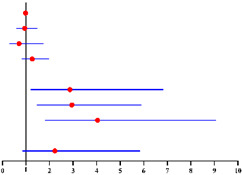	**Adjusted OR ^c^ (95% CI)**	***p*** **^e^**	**Characteristics**	**No.**	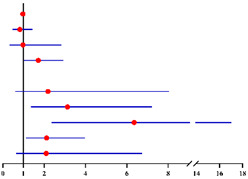	**Adjusted OR ^d^ (95% CI)**	***p*** **^e^**
**Age (years)**:	624		0.99 (0.95–1.02)	0.423	**Age (years)**:	447		0.97 (0.93–1.01)	0.121
**BMI (kg/m²)**: ≥28 vs. <28	621		0.95 (0.60–1.50)	0.813	**BMI (kg/m²)**: ≥ 28 vs. < 28	446		0.82 (0.47–1.43)	0.484
**Diabetes**: Yes vs. No	624		0.71 (0.29–1.75)	0.451	**Diabetes**: Yes vs. No	447		0.97 (0.33–2.85)	0.957
**Hypertension**: Yes vs. No	624		1.26 (0.81–1.98)	0.307	**Hypertension**: Yes vs. No	447		1.72 (1.01–2.92)	**0.046**
**Menopause time (years)**:	624				**Menopause time (years)**:	447			
<2 years vs. No			2.87 (1.20–6.84)	**0.018**	<2 years vs. No			2.19 (0.60–8.03)	0.238
≥2–< 5 years vs. No			2.95 (1.47–5.91)	**0.002**	≥2–<5 years vs. No			3.13 (1.36–7.21)	**0.007**
≥5 years vs. No			4.04 (1.80–9.06)	**0.001**	≥5 years vs. No			6.35 (2.37–17.03)	**<0.001**
					**CA125 (U/ml)**: ≥35 vs. <35	447		2.12 (1.13–3.98)	**0.020**
**FBG (mmol/L)**: ≥7.0 vs. <7.0	607		2.23 (0.85–5.85)	0.104	**FBG (mmol/L)**: ≥7.0 vs. <7.0	435		2.10 (0.65–6.75)	0.214
Final diagnosis					Final diagnosis				
		← AEH EC →					← AEH EC →		

^a^ In all 624 preoperative-AEH patients. ^b^ In 447 preoperative-AEH patients with available serum CA125 value. ^c^ Adjusted for age, BMI, diabetes, hypertension and FBG level. ^d^ Adjusted for age, BMI, diabetes and FBG level. ^e^ Significant difference *p* < 0.05. Notes: ^f^ CA125 was not included into this analysis for only 447 of 624 preoperative-AEH patients had available serum CA125 value. ^g^ This analysis was performed in 447 of 624 preoperative-AEH patients with available serum CA125 value. Abbreviations: AEH, atypical endometrial hyperplasia; EC, endometrial cancer; OR, odds ratio; CI, confidence interval; BMI, body mass index; CA125, cancer antigen 125; FBG, fasting blood glucose.

**Table 5:** Multivariate logistic analysis models on the correlation between menopausal statuses (premenopausal status, postmenopausal time <2 years, ≥2–<5 years and ≥5 years) and concurrent intermediate-high-risk EC in all 190 finally diagnosed EC patients ^a^ and in 130 finally diagnosed EC patients ^b^ with available serum CA125 value.

Multivariate Analysis without Available CA125 ^f^					Multivariate Analysis with Available CA125 ^g^				
**Characteristics**	**No.**	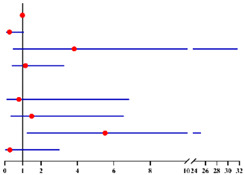	**Adjusted OR ^c^ (95% CI)**	***p*** **^e^**	**Characteristics**	**No.**	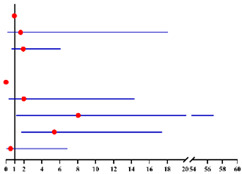	**Adjusted OR ^d^ (95% CI)**	***p*** **^e^**
**Age (years)**:	190		0.97 (0.90–1.05)	0.483	**Age (years)**:	130		0.94 (0.86–1.04)	0.219
**BMI (kg/m²)**: ≥28 vs. <28	189		0.26 (0.07–1.04)	0.057	**Diabetes**: Yes vs. No	130		1.64 (0.15–18.07)	0.686
**Diabetes**: Yes vs. No	190		3.82 (0.46–31.69)	0.214	**Hypertension**: Yes vs. No	130		1.93 (0.61–6.12)	0.266
**Hypertension**: Yes vs. No	190		1.14 (0.38–3.28)	0.806	**Menopause time (years)**:	130			
**Menopause time (years)**:	190				<2 years vs. No			0 (0)	0.999
<2 years vs. No			0.78 (0.09–6.85)	0.821	≥2–< 5 years vs. No			2.00 (0.28–14.35)	0.489
≥2–<5 years vs. No			1.48 (0.34–6.56)	0.604	≥5 years vs. No			8.06 (1.14–56.83)	**0.036**
≥5 years vs. No			5.52 (1.21–25.19)	**0.027**	**CA125 (U/ml)**: ≥35 vs. <35	130		5.40 (1.68–17.41)	**0.005**
**FBG (mmol/L)**: ≥7.0 vs. <7.0	184		0.29 (0.03–3.01)	0.298	**FBG (mmol/L)**: ≥7.0 vs. <7.0	127		0.51 (0.04–6.85)	0.613
Final diagnosis					Final diagnosis				
	← low-risk EC intermediate-high-risk EC →					← low-risk EC intermediate-high-risk EC →			

^a^ In all 190 preoperative-AEH patients finally diagnosed with EC. ^b^ In 130 preoperative-AEH patients finally diagnosed with EC who had available serum CA125 value. ^c^ adjusted for age, BMI, diabetes, hypertension and FBG level. ^d^ adjusted for age, diabetes, hypertension and FBG level. ^e^ Significant difference *p* < 0.05. Notes: ^f^ CA125 was not included into this analysis for only 130 of 190 finally diagnosed EC patients had available CA125 value. ^g^ This analysis was performed in 130 of 190 finally diagnosed EC patients with available serum CA125 value. Abbreviations: EC, endometrial cancer; final-EC, endometrial cancer diagnosed by final histopathology; OR, odds ratio; CI, confidence interval; BMI, body mass index; CA125, cancer antigen 125; FBG, fasting blood glucose. Low-risk EC postoperatively was defined as: endometrioid endometrial cancer grade 1–2, myometrial invasion <50% and no other risk factors presented (which included grade 3, non-endometrioid endometrial cancer, myometrial invasion ≥50%, cervical stromal involvement, extra-uterine involvement or lymph-vascular space invasion). All other endometrial cancer cases were defined as intermediate-high-risk EC.

## Data Availability

The data presented in this study are available on request from the corresponding authors.

## Supplementary Materials

The following are available online at https://www.mdpi.com/article/10.3390/diagnostics12010006/s1: Table S1: Postoperative pathological characteristics and lymph node metastasis of final-EC patients. Table S2: Clinical characteristics of patients diagnosed with low-risk EC and intermediate-high-risk EC by final histopathology. Table S3: The positive and negative predictive values of the combination of “postmenopausal status + CA125 ≥ 35 U/mL” for concurrent EC in final histopathology of 447 preoperative-AEH patients with available serum CA125 value. Figure S1: The distribution of final-EC and final-AEH patients diagnosed postoperatively in 4 subgroups according to menopause years (premenopausal status, postmenopausal time <2 years, ≥2–<5 years and ≥5 years).

## References

[1] Sanderson P.A., Critchley H.O., Williams A.R., Arends M.J., Saunders P.T. (2017). New concepts for an old problem: The diagnosis of endometrial hyperplasia. Hum. Reprod. Update.

[2] Kurman R.J., Kaminski P.F., Norris H.J. (1985). The behavior of endometrial hyperplasia. A long-term study of “untreated” hyperplasia in 170 patients. Cancer.

[3] Erdem B., Asicioglu O., Seyhan N.A., Peker N., Ulker V., Akbayir O. (2018). Can concurrent high-risk endometrial carcinoma occur with atypical endometrial hyperplasia?. Int. J. Surg..

[4] Rakha E., Wong S.C., Soomro I., Chaudry Z., Sharma A., Deen S., Chan S., Abu J., Nunns D., Williamson K. (2012). Clinical outcome of atypical endometrial hyperplasia diagnosed on an endometrial biopsy: Institutional experience and review of literature. Am. J. Surg. Pathol..

[5] Antonsen S.L., Ulrich L., Hogdall C. (2012). Patients with atypical hyperplasia of the endometrium should be treated in oncological centers. Gynecol. Oncol..

[6] Hahn H.S., Chun Y.K., Kwon Y.I., Kim T.J., Lee K.H., Shim J.U., Mok J.E., Lim K.T. (2010). Concurrent endometrial carcinoma following hysterectomy for atypical endometrial hyperplasia. Eur. J. Obstet. Gynecol. Reprod. Biol..

[7] Zaino R.J., Kauderer J., Trimble C.L., Silverberg S.G., Curtin J.P., Lim P.C., Gallup D.G. (2006). Reproducibility of the diagnosis of atypical endometrial hyperplasia: A Gynecologic Oncology Group study. Cancer.

[8] Trimble C.L., Kauderer J., Zaino R., Silverberg S., Lim P.C., Burke J.J., Alberts D., Curtin J. (2006). Concurrent endometrial carcinoma in women with a biopsy diagnosis of atypical endometrial hyperplasia: A Gynecologic Oncology Group study. Cancer.

[9] Karamursel B.S., Guven S., Tulunay G., Kucukali T., Ayhan A. (2005). Which surgical procedure for patients with atypical endometrial hyperplasia?. Int. J. Gynecol. Cancer.

[10] Zhou L., Meng Z., Wu Y., Zhu H., Wang X. (2014). Prediction of endometrial carcinogenesis probability while diagnosed as atypical endometrial hyperplasia: A new risk model based on age, CA199 and CA125 assay. Eur. J. Obstet. Gynecol. Reprod. Biol..

[11] National Comprehensive Cancer Network. https://www.nccn.org/guidelines/guidelines-detail?category=patients&id=41.

[12] Raglan O., Kalliala I., Markozannes G., Cividini S., Gunter M.J., Nautiyal J., Gabra H., Paraskevaidis E., Martin-Hirsch P., Tsilidis K.K. (2019). Risk factors for endometrial cancer: An umbrella review of the literature. Int. J. Cancer.

[13] Lortet-Tieulent J., Ferlay J., Bray F., Jemal A. (2018). International Patterns and Trends in Endometrial Cancer Incidence, 1978–2013. J. Natl. Cancer Inst..

[14] Chi D., Berchuck A., Dizon D.S., Yashar C.M. (2017). Principles and Practice of Gynecologic Oncology.

[15] Vetter M.H., Smith B., Benedict J., Hade E.M., Bixel K., Copeland L.J., Cohn D.E., Fowler J.M., O’Malley D., Salani R. (2020). Preoperative predictors of endometrial cancer at time of hysterectomy for endometrial intraepithelial neoplasia or complex atypical hyperplasia. Am. J. Obstet. Gynecol..

[16] Gungorduk K., Ozdemir A., Ertas I.E., Sahbaz A., Asicioglu O., Gokcu M., Solmaz U., Harma M., Uzuncakmak C., Dogan A. (2015). A novel preoperative scoring system for predicting endometrial cancer in patients with complex atypical endometrial hyperplasia and accuracy of frozen section pathological examination in this context: A multicenter study. Gynecol. Obstet. Investig..

[17] Pecorelli S. (2009). Revised FIGO staging for carcinoma of the vulva, cervix, and endometrium. Int. J. Gynaecol. Obstet..

[18] Zhou B.F., The Cooperative Meta-Analysis Group of the Working Group on Obesity in China (2002). Predictive values of body mass index and waist circumference for risk factors of certain related diseases in Chinese adults: Study on optimal cut-off points of body mass index and waist circumference in Chinese adults. Biomed. Environ. Sci..

[19] Shan W., Ning C., Luo X., Zhou Q., Gu C., Zhang Z., Chen X. (2014). Hyperinsulinemia is associated with endometrial hyperplasia and disordered proliferative endometrium: A prospective cross-sectional study. Gynecol. Oncol..

[20] Dinh W., Lankisch M., Nickl W., Scheyer D., Scheffold T., Kramer F., Krahn T., Klein R.M., Barroso M.C., Futh R. (2010). Insulin resistance and glycemic abnormalities are associated with deterioration of left ventricular diastolic function: A cross-sectional study. Cardiovasc. Diabetol..

[21] Merisio C., Berretta R., De Ioris A., Pultrone D.C., Rolla M., Giordano G., Tateo S., Melpignano M. (2005). Endometrial cancer in patients with preoperative diagnosis of atypical endometrial hyperplasia. Eur. J. Obstet. Gynecol. Reprod. Biol..

[22] Sobczuk K., Sobczuk A. (2017). New classification system of endometrial hyperplasia WHO 2014 and its clinical implications. Prz. Menopauzalny.

[23] McKenney J.K., Longacre T.A. (2009). Low-grade endometrial adenocarcinoma: A diagnostic algorithm for distinguishing atypical endometrial hyperplasia and other benign (and malignant) mimics. Adv. Anat. Pathol..

[24] Bischof P., Tseng L., Brioschi P.A., Herrmann W.L. (1986). Cancer antigen 125 is produced by human endometrial stromal cells. Hum. Reprod..

[25] Takami M., Sakamoto H., Ohtani K., Takami T., Satoh K. (1997). An evaluation of CA125 levels in 291 normal postmenopausal and 20 endometrial adenocarcinoma-bearing women before and after surgery. Cancer Lett..

[26] Grover S., Koh H., Weideman P., Quinn M.A. (1992). The effect of the menstrual cycle on serum CA 125 levels: A population study. Am. J. Obstet. Gynecol..

[27] Nagamani M., Stuart C.A., Doherty M.G. (1992). Increased steroid production by the ovarian stromal tissue of postmenopausal women with endometrial cancer. J. Clin. Endocrinol. Metab..

[28] Kang S., Kang W.D., Chung H.H., Jeong D.H., Seo S.S., Lee J.M., Lee J.K., Kim J.W., Kim S.M., Park S.Y. (2012). Preoperative identification of a low-risk group for lymph node metastasis in endometrial cancer: A Korean gynecologic oncology group study. J. Clin. Oncol..

[29] Soper J.T., Berchuck A., Olt G.J., Soisson A.P., Clarke-Pearson D.L., Bast R.C. (1990). Preoperative evaluation of serum CA 125, TAG 72, and CA 15-3 in patients with endometrial carcinoma. Am. J. Obstet. Gynecol..

[30] Bast R.C., Klug T.L., St John E., Jenison E., Niloff J.M., Lazarus H., Berkowitz R.S., Leavitt T., Griffiths C.T., Parker L. (1983). A radioimmunoassay using a monoclonal antibody to monitor the course of epithelial ovarian cancer. N. Engl. J. Med..

[31] Sood A.K., Buller R.E., Burger R.A., Dawson J.D., Sorosky J.I., Berman M. (1997). Value of preoperative CA 125 level in the management of uterine cancer and prediction of clinical outcome. Obstet. Gynecol..

