# Supplementary material for: Menopausal Status Combined with Serum CA125 Level Significantly Predicted Concurrent Endometrial Cancer in Women Diagnosed with Atypical Endometrial Hyperplasia before Surgery
Source: Diagnostics (Basel). 2021 Dec 21;12(1):6. doi: 10.3390/diagnostics12010006 (PMC8775082; doi:10.3390/diagnostics12010006)
Supplement: Supplementary file 1 [file diagnostics-12-00006-s001.zip › Supplementary Figure S1.pdf]

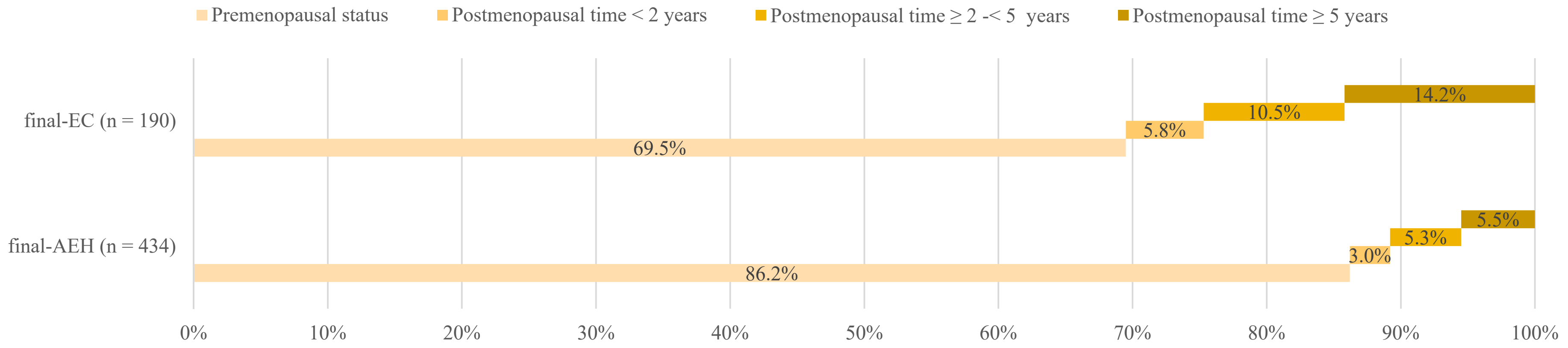


**Figure S1.** The distribution of final-EC and final-AEH patients diagnosed postoperatively in 4 subgroups according to menopause years (premenopausal status, postmenopausal time < 2 years, ≥ 2 -< 5 years and ≥ 5 years). Notes: Subgroups of menopause time were divided as: premenopausal status, postmenopausal time < 2 years, ≥ 2 -< 5 years and ≥ 5 years. Abbreviations: final-AEH: atypical endometrial hyperplasia diagnosed by final histopathology; final-EC: endometrial cancer diagnosed by final histopathology; CA125, cancer antigen 125.
