# Supplementary material for: Menopausal Status Combined with Serum CA125 Level Significantly Predicted Concurrent Endometrial Cancer in Women Diagnosed with Atypical Endometrial Hyperplasia before Surgery
Source: Diagnostics (Basel). 2021 Dec 21;12(1):6. doi: 10.3390/diagnostics12010006 (PMC8775082; doi:10.3390/diagnostics12010006)
Supplement: Supplementary file 1 [file diagnostics-12-00006-s001.zip › Supplementary Table S1.pdf]

Table S1. Postoperative pathological characteristics and lymph node metastasis of final-EC patients.

Characteristic	Patients (*n*)	final-EC (*n* = 190)
**FIGO stage (2009)**	190	
**IA**		170 (89.5%)
**IB**		7 (3.7%)
**II**		6 (3.2%)
**IIIA**		3 (1.6%)
**IIIC1**		1 (0.5%)
**IIIC2**		3 (1.6%)
**Grade**	183	
**1**		172 (94.0%)
**2**		9 (4.9%)
**3**		2 (1.1%)
**Myometrial invasion**	190	
**No invasion**		48 (25.3%)
**< 50% invasion**		131 (68.9%)
**≥ 50% invasion**		11 (5.8%)
**Histology**	190	
**Endometrioid**		184 (96.8%)
**Serous**		1 (0.5%)
**Mucinous**		1 (0.5%)
**Secretory**		4 (2.1%)
**LVSI**	190	
**Absent**		180 (94.7%)
**Present**		10 (5.3%)
**Peritoneal washes**	71	
**(-)**		68 (95.8%)
**(+)**		3 (4.2%)
**Cervical infiltration**	190	
**(-)**		183 (96.3%)
**(+)**		7 (3.7%)
**Parametrium involvement**	190	
**(-)**		190 (100%)
**(+)**		0 (0%)
**MELF**	190	
**(-)**		182 (95.8%)
**(+)**		8 (4.2%)
**Para-aortic lymph node**	16	
**(-)**		13 (81.2%)
**(+)**		3 (18.8%)
**Pelvic lymph node**	37	
**(-)**		34 (91.9%)
**(+)**		3 (8.1%)

Data shown were number (%).

Abbreviations: final-EC, endometrial cancer diagnosed by final histopathology; LVSI, lymph-vascular space invasion; MELF, microcystic, elongated and fragmented.
