# Supplementary material for: Menopausal Status Combined with Serum CA125 Level Significantly Predicted Concurrent Endometrial Cancer in Women Diagnosed with Atypical Endometrial Hyperplasia before Surgery
Source: Diagnostics (Basel). 2021 Dec 21;12(1):6. doi: 10.3390/diagnostics12010006 (PMC8775082; doi:10.3390/diagnostics12010006)
Supplement: Supplementary file 1 [file diagnostics-12-00006-s001.zip › Supplementary Table S2.pdf]

Table S2. Clinical characteristics of patients diagnosed with low-risk EC and intermediate-high-risk EC by final histopathology.

**Characteristics**	**Number**	**Total final-EC (n = 190)**	**low-risk EC (n = 160)**	**intermediate-high-risk EC (n = 30)**	***p* value** [b,c]
		Median (interquartile range)			
Age (years)	190	49 (44-53)	48.5 (45-53)	51.5 (43-59)	0.211
BMI (kg/m^2) [a]	189	24.46 (22.34-27.60)	24.56 (22.43-27.89)	23.74 (21.55-26.71)	0.159
		Number (%)			
BMI (kg/m^2) [a]	189				0.146
< 28		148 (78.3%)	121 (76.1%)	27 (90.0%)	
≥ 28		41 (21.7%)	38 (23.9%)	3 (10.0%)	
Menopausal status	190				0.097
Premenopausal		132 (69.5%)	115 (71.9%)	17 (56.7%)	
Postmenopausal		58 (30.5%)	45 (28.1%)	13 (43.3%)	
Fertility	190				0.218
Pluripara		179 (94.2%)	149 (93.1%)	30 (100.0%)	
Nullipara		11 (5.8%)	11 (6.9%)	0 (0)	
Tubal ligation	190				0.522
NO		175 (92.1%)	146 (91.3%)	29 (96.7%)	
YES		15 (7.9%)	14 (8.8%)	1 (3.3%)	
Diabetes	190				0.724
NO		177 (93.2%)	150 (93.8%)	27 (90.0%)	
YES		13 (6.8%)	10 (6.3%)	3 (10.0%)	
Hypertension	190				0.382
NO		139 (73.2%)	119 (74.4%)	20 (66.7%)	
YES		51 (26.8%)	41 (25.6%)	10 (33.3%)	
FBG (mmol/L)	184				1.000
< 7.0		171 (92.9%)	144 (92.9%)	27 (93.1%)	
≥ 7.0		13 (7.1%)	11 (7.1%)	2 (6.9%)	
HOMA-IR [a]	61				0.490
< 2.95		45 (73.8%)	39 (76.5%)	6 (60.0%)	
≥ 2.95		16 (26.2%)	12 (23.5%)	4 (40.0%)	
CA125 (U/ml) [a]	130				0.003
< 35		109 (83.8%)	96 (88.1%)	13 (61.9%)	
≥ 35		21 (16.2%)	13 (11.9%)	8 (38.1%)	
Sampling method	190				0.613
D&C alone		134 (70.5%)	114 (71.3%)	20 (66.7%)	
D&C with HSC		56 (29.5%)	46 (28.7%)	10 (33.3%)	

Data shown were median (interquartile range) or number (%).

[a] All variables were analyzed among 190 final-EC patients except for BMI, FBG, HOMA-IR and CA125. Missing data included 1 case for BMI, 6 for FBG, 129 for HOMA-IR and 60 for CA125.

[b] p value: difference between low-risk EC group and intermediate-high-risk EC group.

[c] Significant difference $p < 0.05$.

Abbreviations: EC, endometrial cancer; final-EC: endometrial cancer diagnosed by final histopathology; BMI, body

mass index; FBG, fasting blood glucose; HOMA-IR, homeostasis model assessment-insulin resistance; CA125, cancer antigen 125; D&C, dilatation and curettage; HSC, hysteroscopy. Low-risk EC postoperatively was defined as: endometrioid endometrial cancer grade 1-2, myometrial invasion < 50% and no other risk factors presented (which included grade 3, non-endometrioid endometrial cancer, myometrial invasion ≥ 50%, cervical stromal involvement, extra-uterine involvement or lymph-vascular space invasion). All other endometrial cancer cases were defined as intermediate-high-risk EC.
