# Supplementary material for: Menopausal Status Combined with Serum CA125 Level Significantly Predicted Concurrent Endometrial Cancer in Women Diagnosed with Atypical Endometrial Hyperplasia before Surgery
Source: Diagnostics (Basel). 2021 Dec 21;12(1):6. doi: 10.3390/diagnostics12010006 (PMC8775082; doi:10.3390/diagnostics12010006)
Supplement: Supplementary file 1 [file diagnostics-12-00006-s001.zip › Supplementary Table S3.pdf]

Table S3. The positive and negative predictive values of the combination of "postmenopausal status + CA125 ≥ 35 U/ml" for concurrent EC in final histopathology of 447 preoperative-AEH patients [a] with available serum CA125 value.

	Total preoperative-AEH patients (*n*)	Final histopathology		Positive predictive value (%)	Negative predictive value (%)
		final-EC (*n*)	final-AEH (*n*)		
**Postmenopausal status+ CA125 ≥ 35 U/ml**	6	5	1	83.3% (5/6)	-
**Others [b]**	441	125	316	-	71.7% (316/441)
**Total (*n*)**	447	130	317	-	-

Data shown were number or percentage.

[a] In total of 447 patients who had serum CA125 value with available data.

[b] These patients included the preoperative-AEH patients with "premenopausal status + CA125 < 35 U/ml" and "either postmenopausal status or CA125 ≥ 35 U/ml".

Notes: According to menopause status and serum of CA125 level, 447 preoperative-AEH patients were divided into three subgroups: "premenopausal status + CA125 < 35 U/ml" (no risk factor), "either postmenopausal status or CA125 ≥ 35 U/ml" (either one risk factor) and "postmenopausal status + CA125 ≥ 35 U/ml" (both risk factors).

Abbreviations: AEH: atypical endometrial hyperplasia; EC: endometrial cancer; final-AEH: atypical endometrial hyperplasia diagnosed by final histopathology; final-EC: endometrial cancer diagnosed by final histopathology; CA125, cancer antigen 125.
